# Bladder cancer microbiome and its association with chemoresponse

**DOI:** 10.3389/fonc.2025.1506319

**Published:** 2025-07-21

**Authors:** Rashida Ginwala, Laura Bukavina, Mohit Sindhani, Erika Nachman, Suraj Peri, Jodie Franklin, John Drevik, Sarah Christianson, Daniel M. Geynisman, Alexander Kutikov, Philip H. Abbosh

**Affiliations:** ^1^ Fox Chase Cancer Center, Philadelphia, PA, United States; ^2^ Department of Urology, Cleveland Clinic Foundation, Cleveland, OH, United States; ^3^ India Institute of Technology, Delhi, India; ^4^ Department of Molecular Virology and Microbiology, Baylor College of Medicine, Houston, TX, United States; ^5^ Synthesis and Sequencing Facility, Johns Hopkins University School of Medicine, Baltimore, MD, United States; ^6^ Albert Einstein Medical Center, Philadelphia, PA, United States

**Keywords:** bladder cancer, urothelial carcinoma, BBN, microbiome, microbiota, gemcitabine, 16S sequencing

## Abstract

**Background:**

The microbiome is widely known to cause come types of cancer, modify cancer biology, and impact therapeutic efficacy. Despite the urinary microbiome being one of the most clinically significant microbiomes in human health, it is one of the least well-described.

**Methods and materials:**

To begin to annotate the urinary microbiome present in bladder cancers, we analyzed human genome-filtered sequencing data from the Cancer Genome Atlas (TCGA) from 116 tumors (duplicates from 22 tumors), 22 adjacent normal bladder tissues, and 99 blood samples to classify reads originating from known microbiota. We also performed 16S rRNA sequencing on urine samples from 55 patients with urothelial carcinoma and 13 non-cancer patients. Additionally, we compared microbiome from matched urine samples and archival diagnostic tumor samples from 21 bladder cancer patients. For our animal experiments, starting at 8-12 weeks of age, male (n=33) and female (n=22) C57BL/6 mice were administered 0.05% N-butyl-N-(4-hydroxybutyl)-nitrosamine (BBN) in drinking water for 12 weeks then switched to regular water. Control mice drank regular water. Bladders were collected for 16S rRNA sequencing pre-exposure, after 6 and 12 weeks of exposure, and at the time of tumor identification (typically between 14-22 weeks from start of treatment using this model). Finally, because of our findings, we tested the effects of *E.coli* infection on gemcitabine toxicity in bladder cancer cell lines.

**Results:**

Twenty-seven viral and bacterial species were found to be enriched in the tumor samples from TCGA cohort, including sex-specific enrichment of *Lactobacillus* and *Prevotella* in female bladder cancer patients which also are prevalent in the normal female genitourinary tract. We found key differences in urinary microbiota profiles that distinguish cancer patients from healthy control. We also found *Granulicatella* and *Proteus* were enriched in patients who did not respond to neoadjuvant chemotherapy while *E. Faecalis* was enriched in responders. Additionally, we found 32% overlap between microbiota of urine and archival diagnostic tumor samples. Because bladder cancer patients undergoing surgical procedures are exposed to a single dose of peri-procedure antibiotics, we took advantage of a ‘natural experiment’ to measure microbial changes in urology patients receiving a single dose of antibiotics for skin procedures, finding that there are very few changes that persist for 1 month. Additionally, we measured microbial changes during BBN-induced carcinogenesis in a mouse model and observed that changes are more likely related to BBN exposure itself rather than carcinogenesis as changes induced by BBN resolve after BBN withdrawal. Lastly, urine from bladder cancer patients harbored abundant Gammaproteobacteria, which in cell culture experiments, detoxified gemcitabine, a commonly used therapy in bladder cancer.

**Conclusions:**

In summary, we identify many new relationships between the microbiome and bladder cancer that are clinically relevant and lay the groundwork important functional studies in the future.

## 1 Introduction

Although the role of the microbiome in human health and disease has been long established, the depth of this association is only recently coming to the fore due to the advent of culture-independent detection techniques, particularly next generation sequencing and metagenomics analyses (1, 2). Microbial dysbiosis has been associated with several diseases including infectious diseases, metabolic disorders, gastrointestinal (GI) abnormalities, and cancer (2, 3). About 20% of tumors have been linked to associative microbiota that are quite distinct from the surrounding healthy tissue or healthy subjects (1). However, there is yet some debate as to whether these microbial signatures arise as a consequence of the tumor or contribute directly to their genesis or persistence. Among the various cancer microbiomes that have been described, the urinary bladder has now been shown to host an extensive microbial population (4–7). However, a great deal yet is to be revealed about the bladder cancer microbiome in order to explore its potential as diagnostic and prognostic biomarkers or even as pharmaceutical treatments, prebiotics and probiotics.

Bladder cancer is the second-most common of the genitourinary cancers and results in a significant health and economic burden to patients worldwide. Several of the genetic and environmental etiological factors, including cigarette smoking and occupational exposures, have been implicated in influencing the human microbiota (8–10). Thus, it is plausible that alterations to the urinary microbiome may play an important role in the pathogenesis and persistence of bladder cancer and also help shape the response to anti-tumor therapy. There is some evidence of altered microbiome in patients with urothelial carcinoma. For instance, Xu et al. observed an enrichment of *Streptococcus* in urine of 5 of 8 bladder cancer patients compared to healthy subjects (11). In another study, the urine of patients with bladder cancer showed enrichment of the bacterial genera *Fusobacterium*, *Actinobaculum*, *Facklamia*, *Campylobacter*, and *Subdoligranulum* and the family *Ruminococcaceae* while the genera *Veillonella*, *Streptococcus* and *Corynebacterium* were more abundant in urines from healthy individuals (12). A study analyzing urine samples from Chinese male patients with bladder cancer found specific enrichment of the bacterial genera *Acinetobacter, Anaerococcus*, and *Sphingobacterium* and reduction in other bacterial genera (e.g. *Serratia*, *Proteus*, and *Roseomonas*) compared to non-neoplastic controls (13). These differences between the urinary microbiota of bladder cancer patients and healthy individuals may suggest a role of the microbiome in influencing tumor pathogenesis.

Accumulating evidence suggests gender-related differences in the urinary microbiome, likely due to anatomical variations, effects of sex-hormones and metabolism of carcinogenic toxins in the bladder, possibly contributing to the higher prevalence of bladder cancer in men (12, 14). Studies from healthy individuals revealed that the genera *Lactobacillus* and *Gardnerella* predominate in the female urinary tract while *Corynebacterium*, *Staphylococcus*, and *Streptococcus* were abundant in males (15). Notably, the female urinary microbiome was more diversified at the genus level than that of males (11). Additionally, age-related changes to the urinary microbiome have also been reported (6, 16).

However, further studies outlining the cause-effect relationship between the urinary microbiome and bladder cancer tumorigenesis need to be carried out through longitudinal analyses of patient samples at various stages of disease progression. Further, metabolism of certain drugs by urinary microbiota have previously shown to desensitize the tumor to the drug, rendering the therapy ineffective. For example, gemcitabine is frequently used in the systemic treatment of bladder cancer and was shown to be detoxified by *Mycoplasma* spp found in pancreatic tumors (17). Hence, a thorough investigation of the microbial content of bladder cancer patients will help define their role in treatment response and help guide the shaping of an effective treatment regimen.

Despite being the most proximal microbiome to bladder tumors, there still remains much to be elucidated with respect to urine. BCG was the first immunotherapy that was used in the treatment of cancer and is the backbone of the treatment of high grade nonmuscle invasive bladder cancer (18). There is an unmet need of developing more effective and less toxic treatment options for radical cystectomy candidates, and the alteration of the microbiome or microbial therapies like BCG might be employed. Annotation of the bacterial species that associate with complete response to chemotherapy would permit analysis to determine if they are causative of response to chemotherapy, which could lead to development of novel therapeutic strategies. With a view to achieve this, we set out to describe the bladder cancer microbiome and the changes in the urinary microbiome of patients as they undergo neoadjuvant chemotherapy in anticipation of radical cystectomy. Lastly, we treated mice with the well-known murine bladder carcinogen N-butyl-N-(4-hydroxybutyl)-nitrosamine (BBN) and collected bladder tissue at 5 different time points to analyze whether the process of carcinogenesis is associated with changes in the microbiome.

## 2 Methods

### 2.1 Subject recruitment and sample collection

Urine specimens were collected from two cohorts of patients. The first cohort of 76 patients had urothelial carcinoma and was enrolled into a prospective clinical trial at Fox Chase Cancer Center to assess the accuracy of cystoscopic evaluation in predicting tumor stage compared to stage at the time of radical cystectomy (NCT02968732, “The pT0 Trial” (19)). Patient characteristics have been listed in **Table 1**. All participants gave their informed consent for collection of urine samples and the subsequent analyses for research purposes. Inclusion/exclusion criteria have been previously described.

**Table 1:** Patient demographics of subjects enrolled into the pT0 Trial.

Patient Characteristics	
Gender	
Male	62
Female	14
Age (years)	
Median	70
Range	42-84
Race	
Caucasian	72
Black or African-American	4
Stage	
1	21
2	31
3	19
4	3
Neoadjuvant Chemotherapy	
None	23
AMVAC	42
Gemcitabine+Cisplatin	5
Other	6
Intravesical therapy	
None	41
BCG	27
MMC	2

The second cohort was enrolled from outpatient clinics from Albert Einstein Medical Center. Patients provided informed written consent to an IRB-approved specimen collection protocol (2019-88). Urine was collected from patients undergoing benign, nonendoscopic general urological procedures such as vasectomy, circumcision, hydrocelectomy, wart removal, etc., at 4 time points: at the time of consultation, at the time of procedure (prior to administration of any antibiotics), at the time of wound check (typically ~1 month), and at the time of follow up (typically ~3 months). Not all patients followed up at all 4 time points.

Clean-catch midstream urine was collected in sterile containers and kept at 4°C until processing within 1-2 h of sampling as per below.

### 2.2 Sample processing and DNA extraction from urine

Urine samples were processed immediately upon receipt. Urine was centrifuged at 4°C at 4000 rpm for 5 min and separated into supernatant and pellet. The pellet was then washed with phosphate buffered saline (PBS) at >10,000 rcf in a microfuge for 3 min. The pellet was frozen at -80°C for DNA extraction at a later time.

DNA was extracted from thawed pellets using a combination of mechanical, thermal and enzymatic disruption techniques. Briefly, the urine pellets were resuspended in a lysis buffer containing 500mM sodium chloride, 50mM Tris-HCl (pH 8.0), 50mM EDTA, and 4% sodium dodecyl sulfate. To this, 50 µl lysozyme (10 mg/ml, Sigma-Aldrich), 6 µl mutanolysin (25 KU/ml, Sigma-Aldrich), and 3 µl lysostaphin (4000 U/ml, Sigma-Aldrich) were added along with 0.4g of sterile zirconia beads (Benchmark Scientific Inc, Sayreville, NJ). Samples were then homogenized in a Mini-BeadBeater™ (BioSpec Products, Bartlesville, OK) at maximum speed for 3 min. Homogenized samples were further incubated at 37°C for 45 min with periodic gentle shaking every 5 min. Subsequently, the samples were heated for 30 min at 70°C. Samples were then centrifuged at 4°C for 5 min at 16,000 rcf. The resulting supernatant was collected, and the pellet was resuspended in lysis buffer for a second round of lysis. The pooled supernatant was then subjected to acetate/isopropanol DNA precipitation with glycogen, washed with 70% ethanol, air dried, resuspended, and stored at -20°C. We found that this approach gave the most consistent 16S profile when compared head-to-head with two commercial kits when applied to homogenize mouse stool pellets.

### 2.3 DNA extraction from trans urethral resection of bladder tumor samples

5-10 micron-thick sections of diagnostic TURBT formalin-fixed paraffin-embedded (FFPE) tissue specimen were prepared. These slides were stored at 4°C until DNA extraction for 16S analysis. Using a fresh sterile blade for each sample, FFPE sections were cut. Mineral oil was used to deparaffinize the tissue. QIAamp DNA FFPE kit (Qiagen) was used for DNA isolation. The aqueous phase (lower, uncolored phase) was transferred to a tube containing sterile zirconia beads and the solution was homogenized in the Mini-BeadBeater™ for 10 minutes. To this, an enzyme cocktail consisting of 50 µl lysozyme (10 mg/ml, Sigma-Aldrich), 6 µl mutanolysin (25 KU/ml, Sigma-Aldrich), and 3 µl lysostaphin (4000 U/ml, Sigma-Aldrich) was added and the tubes were transferred to a water bath set at 37°C for 30 minutes. The supernatant from this step was then taken through the rest of the manufacturer’s protocol to obtain DNA, which was then quantified and used for 16S.

### 2.4 Shotgun sequencing

Post-chemotherapy urine samples from 7 patients on the RETAIN cystectomy avoidance trial (NCT02710734) (20) were used to isolate DNA as described. The takara Thru Plex DNA HV kit was used to generate low pass shotgun sequencing data using 100PE Illumina chemistry. Whole-Genome Sequencing (WGS) Analysis was performed on samples to characterize microbial composition and validate findings from 16S rRNA sequencing. Raw sequencing reads in.fastq format were subjected to quality filtering and adapter trimming using Fastp to remove low-quality bases and short reads. High-quality reads were classified using Kraken2, a k-mer-based taxonomic classifier, against the Standard-Full RefSeq database, which includes archaea, bacteria, viruses, plasmids, human, and UniVec_Core sequences. Kraken2 reports were processed using KrakenTools to extract relevant taxonomic assignments and generate summarized classification reports. Multiple Kraken2 reports were combined to create a final aggregated report, ensuring comprehensive microbial classification across all samples. Species-level identification was performed to determine the relative abundance of microbial taxa. Decontamination was conducted to remove potential human-derived sequences and environmental contaminants from the final dataset.

### 2.5 Assessment of contamination from various sources

To account for bacterial contamination from the laboratory environment, we included negative controls at each stage of the procedure. Contamination during DNA extraction was accounted for by including extraction from a water blank. Impurities introduced at the PCR stages were accounted for by including non-template controls. As positive control for DNA extraction, we collected 12 fresh mouse stool pellets, pooled/rehydrated/homogenized/aliquoted them, into microcentrifuge tubes, and stored at -80°C. One aliquot was used during each batch of DNA extraction. As a second positive control at the PCR stage, we included DNA extracted from the first mouse stool pellet. Both positive controls were carried through all subsequent stages of analysis to ensure reproducibility and identify significant deviations (i.e. batch effects). For the microbiome analysis of TURBT samples, to control for contamination introduced during the paraffin embedding and formalin fixing process, we processed empty paraffin from the edges of the FFPE specimens as negative controls. These were carried through the entire analysis process and any reads detected from these specimens were identified as contamination and excluded from the reads originating from the test samples.

### 2.6 Mouse studies

Mice were studied under an IACUC approved protocol (20-09). Specific pathogen free C57B6 mice (n=56; 33 male, 23 Female) were purchased from Taconic and housed in a specific pathogen free environment. At age 8 weeks, mice were treated with 0.1% BBN in drinking water or regular drinking water from the same source ad libitum. Cages were randomized as to which treatment the cage received, and male and female mice were housed separately. At 6 and 12 weeks, mice were randomly chosen from each cage for euthanasia by C0_2_ inhalation. Bladder tissue was collected and split into two parts. One part was subjected to DNA extraction (described below) and the other part was kept at -80°C. Mice were imaged using µCT excretory urography for the presence of tumors starting at week 14, and when tumors were identified (between 16-22 weeks), mice were sacrificed for bladder collection.

Animals were euthanized according to our IACUC approved (#20-09) protocol by slowly uptitrating the amount of inhaled CO2 until spontaneous respirations stopped. 100% C02 was flowed into an enclosed cage at 1L/min and the flow rate was increased every 20-30 seconds until spontaneous respiration halted for >60 seconds. Cervical dislocation was then used to ensure expiration”.

DNA was isolated from bladder tissue first using proteinase K digestion, then with enzymatic cocktail (mutanolysin, lysostaphin, lysozyme) as described above. DNA was then isolated using phenol chloroform extraction/ethanol precipitation. Pelleted DNA was quantitated as below.

### 2.7 DNA yield assessment

The quantity of the purified genomic DNA from both urine and fecal samples was estimated with the Qubit dsDNA high-sensitivity assay (ThermoFisher Scientific, Waltham, MA) using the Qubit 4 Fluorometer.

### 2.8 16S rRNA library preparation and sequencing

DNA isolated from urine or fecal samples was subjected to 16S PCR amplification using primers for the V3-V4 regions of the bacterial 16S rRNA in two stages. To ensure accurate base-calling on Illumina platforms and reduce the need for phi X DNA, we introduced sequence diversity using a series of frameshifts in the 16S amplicons between the adapter and the V3 or V4 annealing sites in the first stage. This concept was described and validated previously (21). List of 16S PCR amplicon primers and indexing primers can be found in **Supplementary Table 1**. Briefly, 10x PCR buffer, AccuPrime™ Pfx DNA Polymerase (ThermoFisher Scientific), nuclease-free water, a unique pair of frameshift Stage I primers, and 20 ng template DNA were mixed together and amplified in using the conditions listed in **Table 2** Stage I. Following bead-clean up at a ratio of 1.25:1 (PCR product: AMPure XP beads), stage 2 indexing PCR was carried out under the conditions listed in **Table 2** Stage II. Bead-clean up using beads at a ratio of 1:1.12 (PCR product: Ampure beads) was performed after which the purified libraries were quantified using the Qubit 4 Fluorometer and validated on a Tapestation 2200. Libraries were pooled together at equimolar ratio to create a 4nM pool which was then denatured with NaOH following Illumina’s 16S Metagenomic Sequencing Library Preparation protocol for Library Denaturing and MiSeq Sample Loading. Library was spiked with 1-2% PhiX (10 pmol) before sequencing on Illumina MiSeq platform with paired-end 300bp reads. Sequencing was performed at the Johns Hopkins Sequencing and Synthesis facility.

**Table 2:** PCR conditions for 16S metagenomic library preparation.

Cycling conditions	
**Stage 1 PCR**	
95°C for 2 minutes	
10 cycles of	95°C for 15 seconds 64.1°C for 15 seconds 68°C for 40 seconds
	
15 cycles of	95°C for 15 seconds 59.1°C for 15 seconds 68°C for 40 seconds
Hold at 4°C	
	
**Stage 2 PCR**	
95°C for 2 minutes	
8 cycles of	95°C for 15 seconds 55°C for 15 seconds 68°C for 1 minute
Hold at 4°C	

### 2.9 16S rRNA analysis

Detailed description of this analysis pipeline has been previously published (25). Filtered sequences with >97% identity were clustered into Operational Taxonomic Units (OTUs) and classified at the genus against the SILVA 16S ribosomal RNA sequence database (release 138.1). The relative abundance of each OTU was determined for all samples. QIIME 2 platform was utilized for processing and visualizing microbiome data.

### 2.10 Statistical analyses

Alpha diversity metrics between the various groups were compared using Wilcoxon rank-sum or Mann-Whitney (MW) test. Adjustments for multiple comparisons were done using the false-discovery rate (FDR) method at an α level of 0.05. Differentially abundant OTUs were identified using LEfSe (linear discriminant analysis (LDA) effect size) for each pairwise comparison of groups. All analyses were conducted in R and Python.

### 2.11 Cell culture experiments

J82, SW780, and UMUC3 cell lines were used, as well as a cell line that we derived from a mouse that was treated with BBN and developed a tumor. The tumor was dissociated and grown as a monolayer in DMEM+10% FBS. No antibiotics were used in maintenance media unless otherwise specified. Cells were grown in humidified 5% CO_2_ and were checked for *Mycoplasma* approximately every 3 months using PCR-based detection as described previously (22).

RFP-labeled UTI89 is a uropathogenic strain of *E.coli* (UPEC) and was a kind gift from Molly Ingersoll PhD (Institut Pasteur, Paris France). Fresh cultures were grown in LB and CFU/mL were quantitated by streaking agar plates, while storing the undiluted liquid culture at 4°C. Once the titer was calculated, the culture was diluted to 1M CFU/mL and stored in 1 mL aliquots 15% sterile glycerol/LB at -80°C. One week later, the number of CFU was assessed by streaking agar plates in triplicate to adjust for any loss of viability during cryopreservation. This approach provided a true “expected” live bacterial count to use in later experiments.

For all infections, cell lines were infected at a multiplicity of infection of 500. Plates were centrifuged in a swinging bucket rotor at 1000 rcf for 10 min at room temperature to increase bacterial uptake by concentrating them at the bottom surface of the dish. Following centrifugation, the bacteria were allowed to grow intracellularly for 30 min at 37°C. Then, media was exchanged for fresh media containing gentamicin at 50µg/mL, which is bactericidal in the media but does not penetrate into the cells.

Cells were also imaged using the Leica confocal microscope in the Biological Imaging Facility of Fox Chase using Hoechst 33342 to localize the nuclei and phalloidin to image cell cytoplasm. Briefly, cell lines were seeded in the wells of 8-well chamber slide at a density of up to 10,000 cells per well and left to attach for 24 hours. Next day, cells were infected as described previously. Post infection cells were fixed with 4% PFA. This ensured that the bacteria were actually inside the cells and not simply attached to the cell membrane.

Cell TiterGlo was used to estimate the concentration of gemcitabine that inhibits growth by 50% (GI_50_). Briefly, 5,000 to 10,000 cells were plated into each well of a black 96 well dish the night before treatment with 0 to 1000 nM concentrations of gemcitabine. Cell TiterGlo was added to the media per manufacturer’s instructions 3 days later and the GI_50_ was estimated using Graphpad Prism. For determining the effect of UPEC on gemcitabine toxicity, Cell TiterGlo was performed with cells infected with UPEC as described earlier.

Additionally, a colony formation assay was performed to test the toxicity of gemcitabine in the presence of UPEC. 10,000 cells were plated in wells of 24-well plates and allowed to attach for 24 hours prior to infection. On day 2, half the plate was infected with UPEC as shown earlier. Cells were then treated with 0.5x, 1x, and 2x gemcitabine IC50 dose and plates were incubated for 4 hours (J82,BBN,SW780) or 24 hours (UMUC3) after which, gemcitabine-containing media was removed, and fresh media was added. On day 5, media was removed, and cells were washed with PBS. Wells were then stained with 0.25% crystal violet stain (20% methanol) for 30 minutes. The stain was removed, and wells were thoroughly rinsed with water. Plates were allowed to dry o/n then imaged.

To assess the effect of UPEC on gemcitabine metabolism, we performed HPLC and mass spectrometry analysis. Briefly, UPEC was grown to an OD_600_ of about 1 in LB media. Gemcitabine was added to 200 µl of bacterial sample at a concentration of 5x GI_50_ in 5ml of cell culture media. UPEC and gemcitabine were incubated for 1 h at 37°C, after which the samples were centrifuged for 15 mins. The resulting supernatant was filtered using a 0.2µM PES filter. 200 µl of this supernatant was mixed with 800 µl methanol in a pre-chilled tube, vortexed and incubated at -80°C overnight. The next day these samples were centrifuged at 13,000 rpm at 4°C for 15 mins. The resultant supernatant was stored in cryovials at -80°C till the time of analysis at the Wistar Metabolomics and Proteomics Institute.

## 3 Results

### 3.1 Differential enrichment of microbes in MIBC vs normal adjacent tissue

**Figure 1:**
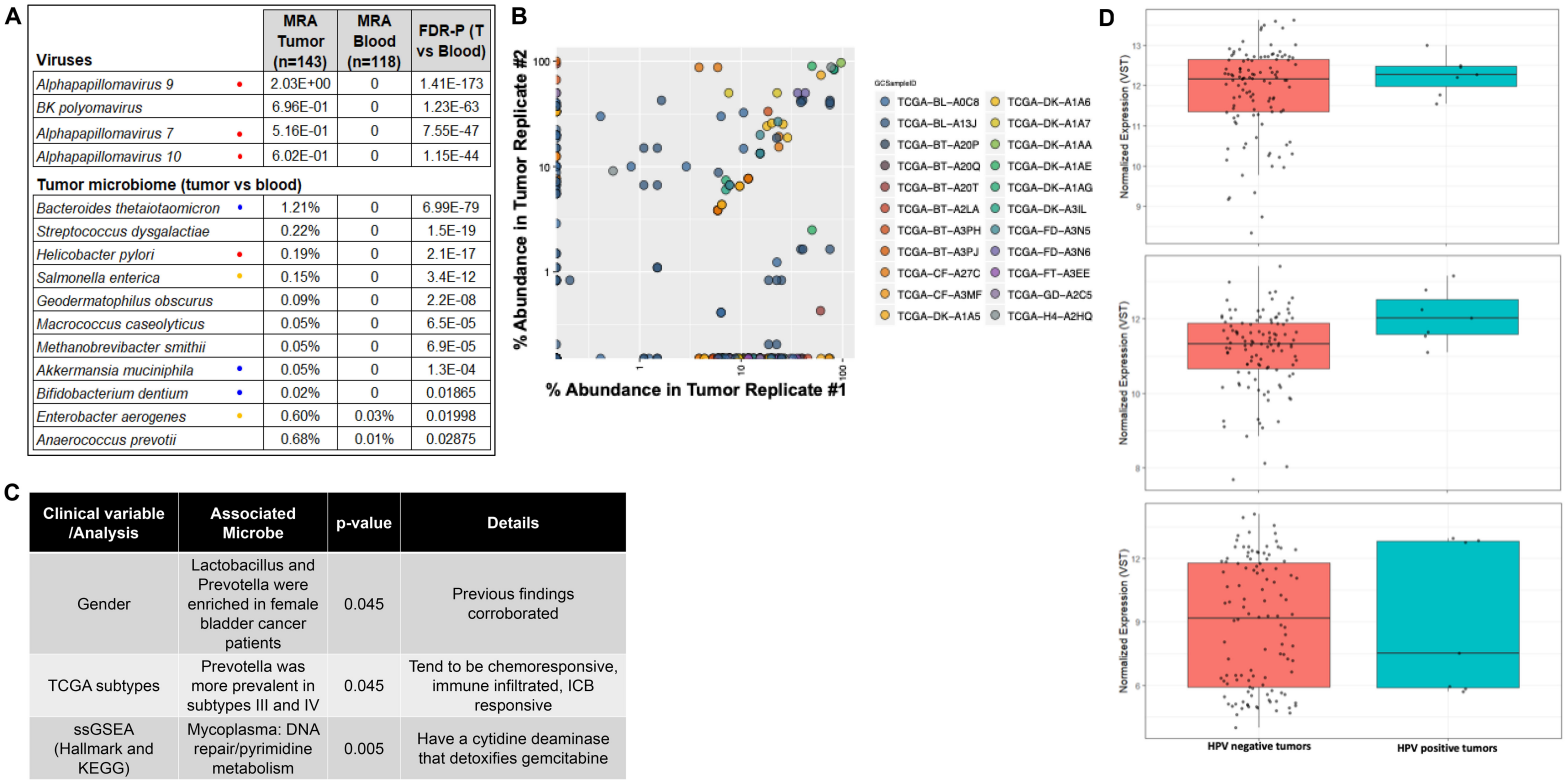
Analysis of TCGA data from bladder cancer cohort. **(A)** Viruses and bacteria were identified using Kraken. MRA, mean relative abundance; FDR-P, FDR-adjusted P value; T=tumor, known cancer-causing organism; = associated with response to immune checkpoint blocking therapies. = can detoxify gemcitabine. **(B)** Pearson analysis of OTUs from available replicates from TCGA samples were cross-validated to each other as a test of reproducibility. **(C)** Table listing specific taxa associated with clinical variables, tumor microenvironment and metabolic pathways. **(D)** mRNA expression levels of TP53, RB1 and CDKN2A demonstrating their gene expression profiles in HPV-positive versus HPV-negative tumors.

Microbial sequences were filtered out from TCGA data from the BLCA cohort and analyzed using Kraken (23), which provides comprehensive classification of metagenomics sequences for identification of microbial and viral members by aligning short reads to bacterial genomic databases. We analyzed 116 tumors, 22 adjacent normal tissues, and 99 blood samples. Mean relative abundance of each OTU in tumor tissue was normalized against all blood samples or all matched adjacent normal tissues to identify taxa that were enriched or depleted. The same analysis was performed to compare adjacent normal vs blood. Several known bladder pathogens were enriched compared to blood including *Enterococcus* spp, *Escherichia coli*, and *Prevotella melananogenica*, in addition to viruses reported by TCGA (24). There were 27 taxa in all, engendering confidence in the approach. The most relevant species have been listed in **Figure 1A**. Thirty-two species were enriched when considering the tumor plus adjacent normal tissue compared to blood denoting a possible ‘microbial field defect’ in the malignant bladder. Notably, bacterial species such as *B. thetaiotamicron*, *A. mucinaphila* and *Bifidobacterium* spp. that have been previously associated with a positive response to anti-CTLA4 (25) and anti-PD-L1 (26) therapies when found in the stool of cancer patients and preclinical models were enriched in tumors. Further, the presence of Alphapapilloma viruses HPV16 and HPV18, the causative agents of cervical cancer (27, 28), were enriched in tumors but not normal bladder. Differential expression of the TP53 (*p*=0.56), RB1 (*p*=0.07) and CDKN2A (*p*=0.72) was seen between HPV positive and negative tumors, however, due to the low number of HPV positive tumors, this difference was not pronounced (**Figure 1D**).

Because TCGA data includes replicate sequencing data for some tissue samples, available replicates were cross validated to each other as a test of reproducibility. A total of 22 tumors had duplicate whole genome sequencing. If a taxon was present with at least 5% abundance in any replicate, we determined its concordance in replicates. Some tumors had triplicate samples, in which three case pairs were evaluated. In total, 119 of 287 taxa were concordant (R=0.33, p=1x10^-8^, Pearson; **Figure 1B**), suggesting there is a true underlying microbiome in this data, and it can be reproducibly detected.

### 3.2 Specific bacterial taxa associated with clinical variables and anti-tumor response

As described in previous reports the urinary microbiome differs between males and females (6). We therefore determined if there was a prevalence of sex-specific taxa in the TCGA data from the bladder cancer patients. We found that *Lactobacillus* and *Prevotella* were enriched in female bladder cancer patients (15% vs 4%, p=0.02; 16% vs 4%, p=0.045 respectively, Fisher’s test). These genera are prevalent in the normal female genitourinary tract (6), supporting the validity of the approach. We also saw that *A. prevotii* and *F. magna* were enriched in females (15% vs 4%; p=0.045).

We leveraged RNAseq data from TCGA samples from which we made tumor microbiome calls to determine if specific genera were associated with intrinsic bladder cancer subtypes (24, 29–31). We found that *Prevotella* was more prevalent in TCGA subtypes III and IV (0%, 3%, 16% and 18%, in subtypes I, II, III, and IV, respectively, p=0.045, Fisher’s test). Finally, RNAseq data was used in single sample gene set enrichment analysis (32) using the Hallmark (33) and KEGG gene sets to show the association of *Mycoplasma* with the Hallmark DNA repair gene set and with the KEGG pyrimidine metabolism pathway (**Figure 1C**). *Mycoplasma* and other Gammaproteobacteria found in pancreatic cancers was recently shown to metabolize gemcitabine, a pyrimidine nucleoside, using a long isoform of cytidine deaminase (17), and it is possible that this enzyme has additional unrecognized effects on DNA repair or pyrimidine metabolism.

**Figure 2:**
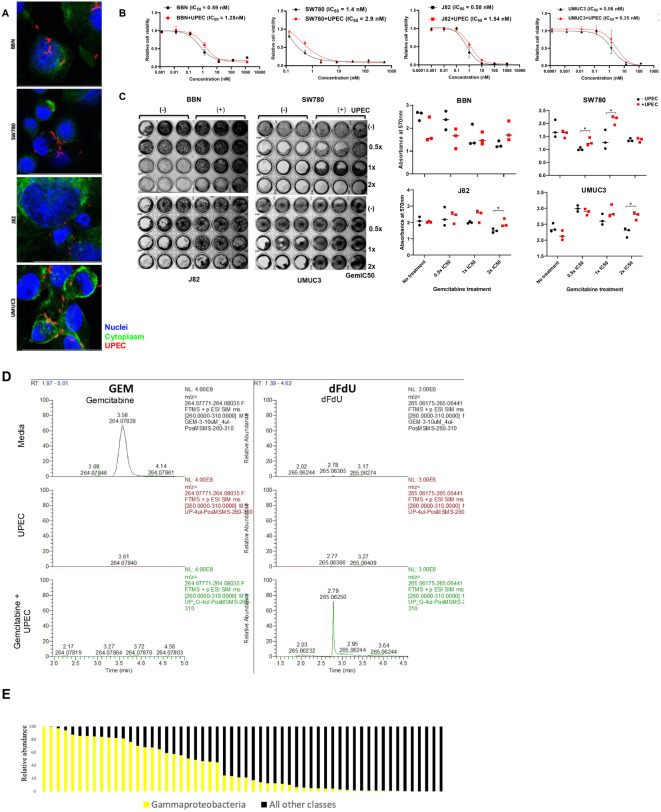
Effect of UPEC on gemcitabine cytotoxicity: **(A)** Cell micrographs depicting infection with E.coli. Hoechst = Nuclei; Phalloidin = Cytoplasm; UPEC **(B)** Cytotoxicity of gemcitabine was tested in 4 cell lines BBN, SW780, J82, and UMUC3 in the presence (red) and (absence) of infection with UPEC. IC50 values were calculated using GraphPad Prism. Data are expressed as mean ± SD from a representative of three independent experiments. **(C)** Results from colony formation assay in BBN, SW780, J82, and UMUC3 analyzing the colony forming ability of the cell lines when treated with 0.5x, 1x, or 2x IC50 concentrations of gemcitabine in the presence or absence of UPEC infection. On the right, crystal violet stain was dissolved and the resultant absorbance was measured at 600nm to quantify the results from the colony forming assay. Statistical significance was determined by Student’s t-test. (*p<0.05). **(D)** HPLC analysis of cell culture media containing gemcitabine alone (top), UPEC alone (middle) and gemcitabine incubated with UPEC for 1 hr (bottom). Gemcitabine (column 1) and dFdU (column 2) peaks were detected. **(E)** Mean relative abundance of gammaproteobacteria or other species in urine samples from bladder cancer patients prior to radical cystectomy. Each column represents a unique urine sample from an individual patient.

Several GU tract pathogens are Gammaproteobacteria, such as *E.coli*, *P. mirabilis*, *P. aeruginosa*, *Klebsiella* spp, and *E. cloacae*. Gemcitabine is a frequently used systemic or local/intravesical therapy in patients with bladder cancer. We therefore set out to determine if gemcitabine metabolism by Gammaproteobacteria would affect gemcitabine toxicity in bladder cancer. We first established that *E.coli* would enter cells by infecting SW780, J82, UMUC3, and our BBN-induced tumor derived cell line (‘BBN’; **Figure 2A**). Spinfected cells were then imaged using a confocal microscope to ensure that bacteria were able to enter into the cells. We next exposed 4 bladder cancer cell lines to increasing doses gemcitabine in 96 well dishes with or without exposure to the RFP-labeled UTI89 strain of *E.coli* to calculate the 50% growth inhibitory concentration (GI_50_) using Cell TiterGlo. This showed that uropathogenic strains of *E.coli* did indeed result in lower efficacy of gemcitabine (**Figure 2B**).

Once GI_50_ were set, each of the 4 cell lines was exposed to 0.5x, 1x, or 2x the GI_50_ with or without UTI89 for colony formation assays. Colony forming ability of the cell lines after treatment with gemcitabine was restored in the presence of UPEC infection indicating loss of gemcitabine toxicity (**Figure 2C**). HPLC analysis further confirmed gemcitabine metabolism to its less-toxic metabolite 2′,2′-difluoro-2′-deoxyuridine (dFdU) when incubated together with UPEC (**Figure 2D**).

### 3.3 Cancer specific alterations in urine microbiome compared to non-cancer controls

**Figure 3:**
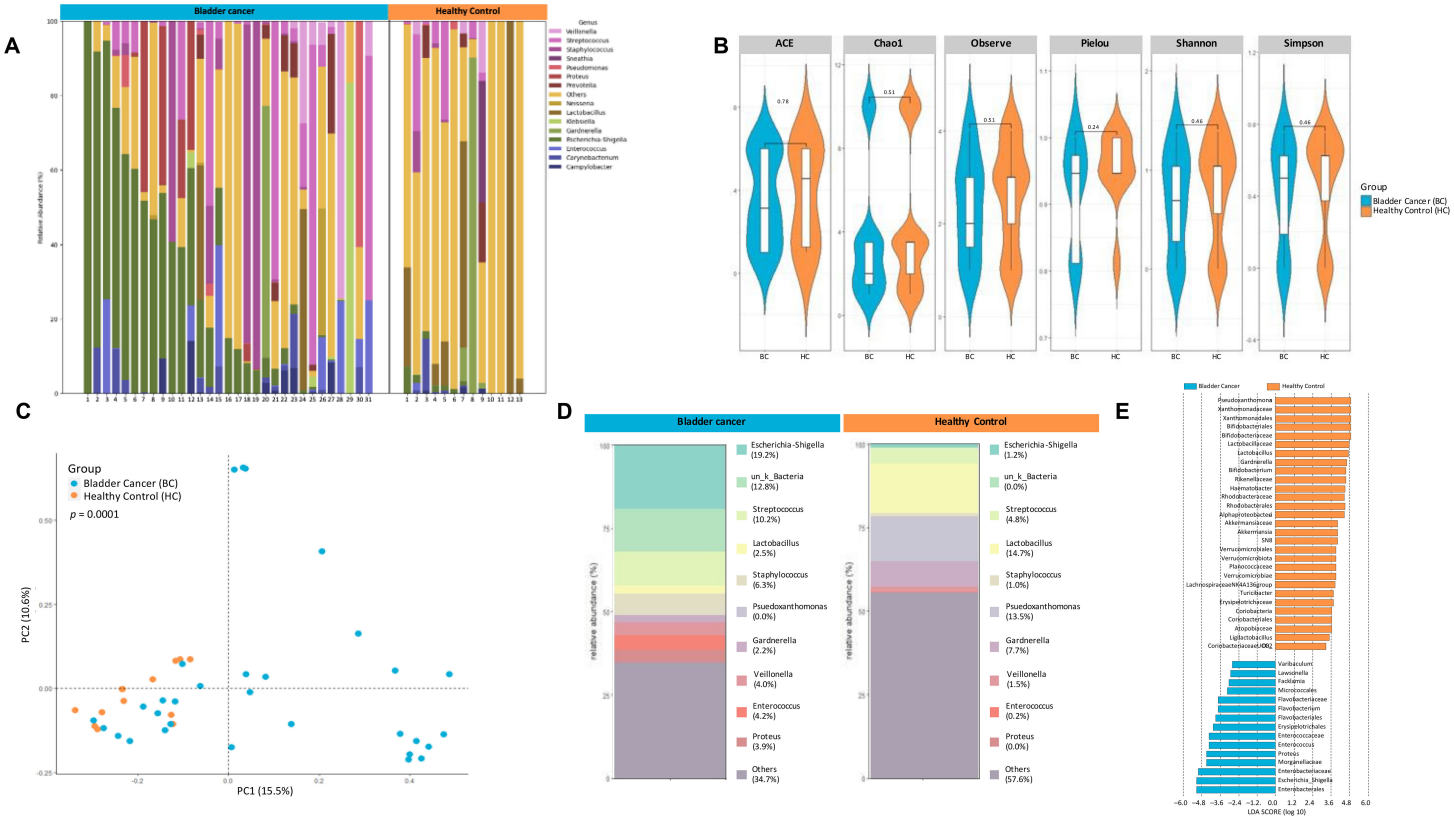
16S metagenomic analyses of urine pellets from bladder cancer patients and non-cancer controls. **(A)** A stacked bar plot showing bacterial composition at the genus level across samples from bladder cancer patients and non-cancer controls. **(B)** Genus-level alpha diversity plots of the urine microbiome from both groups with error bars representing variability in diversity scores **(C)** PCoA using weighted unifrac distances of beta diversity stratified by disease state (ADONIS; Bray-Curtis; *p*=0.0001) **(D)** Bar plot showing bacterial composition at the genus level. Relative abundance is plotted for taxa from each group. **(E)** Histogram of LDA scores for differentially over-abundant taxa in urine samples from bladder cancer (blue bars) and non-cancer controls (orange bars).

The 16S metagenome of urine samples collected from bladder cancer patients (n=31, urine samples obtained prior to initiating chemotherapy) was compared to that of non-bladder cancer controls (N=13; **Figure 3A**).There were no significant differences in typical measures of alpha diversity (**Figure 3B**). However, a clear difference in the microbial community structure as measured by principal components using weighted unifrac distances was observed between the bladder cancer and non-cancer controls (**Figure 3C**). Relative abundance of taxa were distinct between the two groups as seen in **Figure 3D**. As shown using the linear discriminant analysis tool LEfSe, there was an enrichment of 15 OTU including *Enterobacteriales*, *Flavobacterium*, *Varicubaculum*, *Facklamia* in bladder cancer. There was also differential abundance 29 OTU in healthy controls including *Pseudoxanthomonas*, *Gardnerella*, *Bifidobacteriales*, *Lactobacillus*, *Verrucumicrobiales* (**Figure 3E**).

### 3.4 Changes in urinary microbiome diversity in bladder cancer patients after chemotherapy

**Figure 4:**
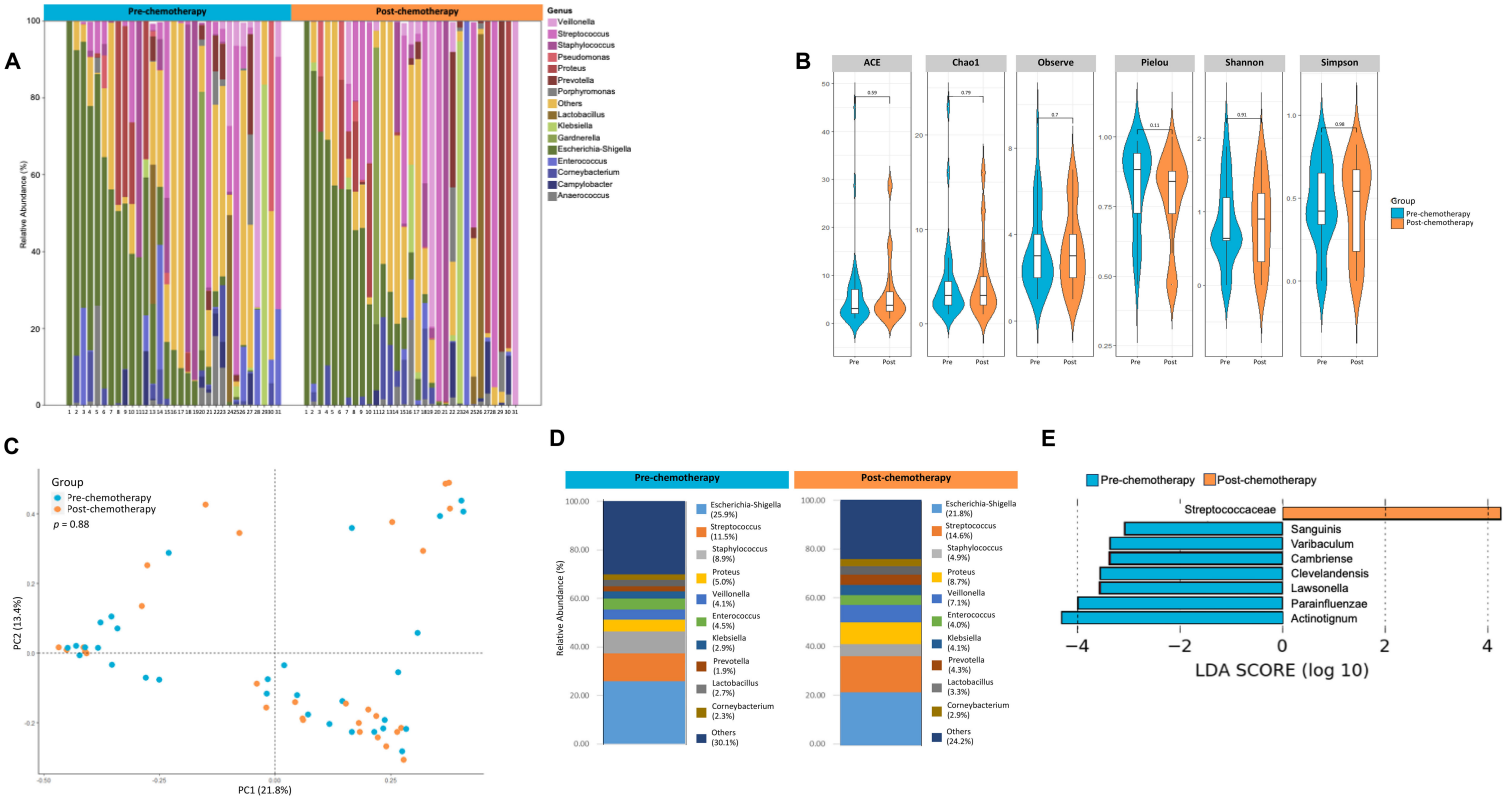
Analysis of urine microbiome differences in bladder cancer patients before and after receiving chemotherapy. **(A)** A stacked bar plot showing bacterial composition at the genus level across samples from bladder cancer patients pre- and post-chemotherapy. **(B)** Genus-level alpha diversity plots of the urine microbiome from both groups with error bars representing variability in diversity scores **(C)** PCoA using weighted unifrac distances of beta diversity stratified by treatment (ADONIS; Bray-Curtis; *p*=0.88) **(D)** Bar plot showing bacterial composition at the genus level. Relative abundance is plotted for taxa from each group. **(E)** Histogram of LDA scores for differentially abundant taxa in urine samples from bladder cancer patients pre- (blue bars) and post- (orange bars) chemotherapy.

Not much is known in terms of the effect of neoadjuvant chemotherapy on the urinary microbiome. In the pT0 Trial, some patients received neoadjuvant chemotherapy, and in these cases, urine samples were obtained both pre- and post- administration (See **Table 1** for details of chemotherapy). Based on alpha diversity metrics and weighted unifrac distances using principal components, no significant difference was seen in the microbial diversity and composition post chemotherapy (**Figures 4A–C**). Relative abundance of taxa at genus level were distinct between urine samples taken pre- and post- chemotherapy (**Figure 4D**). Individually, the patient profiles of microbial composition pre- and post-neoadjuvant chemotherapy show differences in the types of bacterial communities present at the genus level (**Supplementary Figure 1**). LDA scores revealed differential enrichment of *Streptococcaceae* in patients post chemotherapy vs *Lawsonella*, *Varibaculum*, *Sanguinis* prior to chemotherapy (**Figure 4E**).

### 3.5 Differences in urine microbiome between chemo-responders and nonresponders

**Figure 5:**
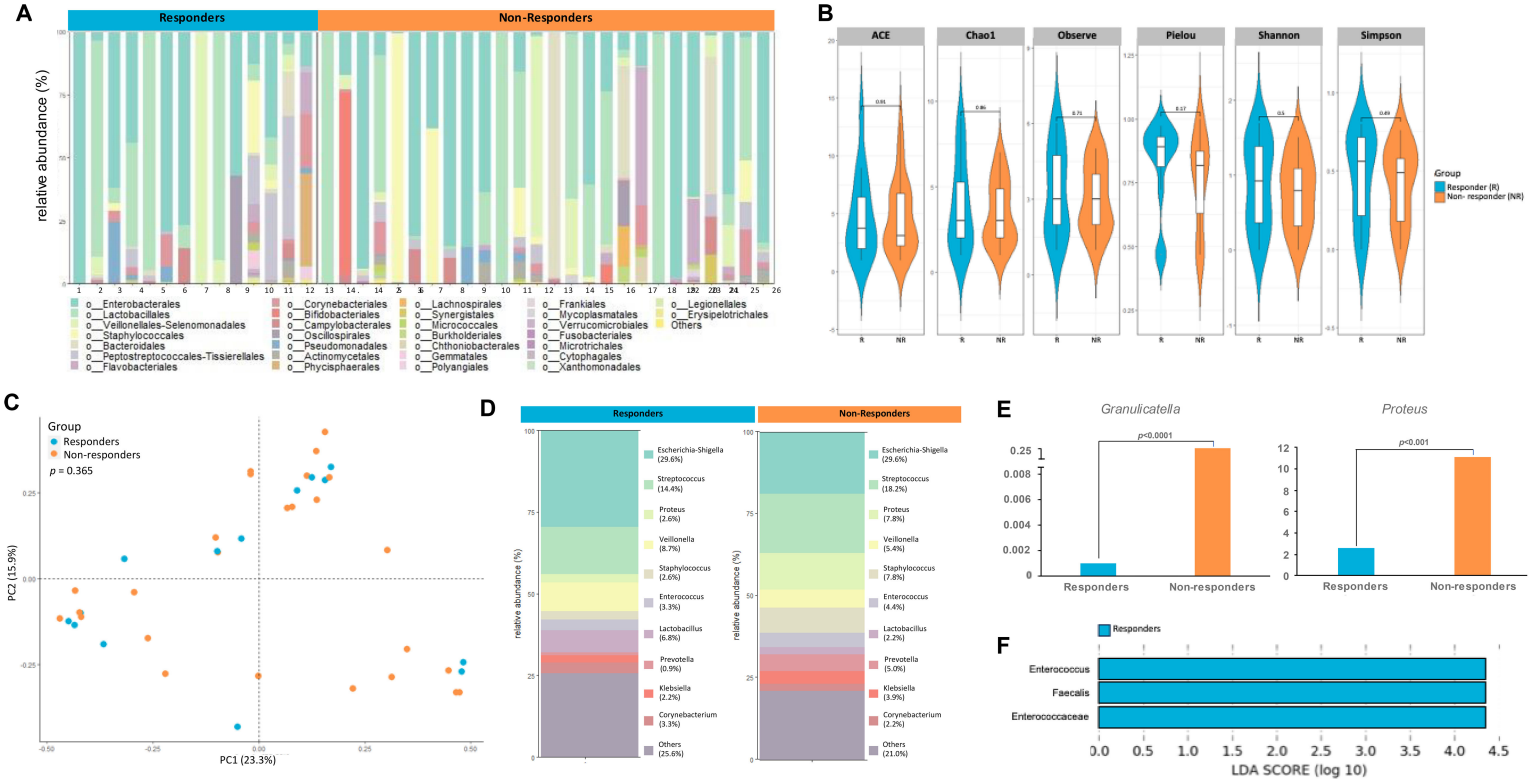
Differences in urine microbiome between responders and non-responders to chemotherapy. **(A)** A stacked bar plot showing bacterial composition at the genus level across samples from responders and non-responders to chemotherapy **(B)** Genus-level alpha diversity plots of the urine microbiome from both groups with error bars representing variability in diversity scores **(C)** PCoA using weighted unifrac distances of beta diversity stratified by response to chemotherapy (ADONIS; Bray-Curtis; *p*=0.365) **(D)** Bar plot showing bacterial composition at the genus level. Relative abundance is plotted for taxa from each group. **(E)** Bar plots showing differential expression of taxa at genus level between responders and non-responders **(F)** Histogram of LDA scores for differentially over-abundant taxa in urine samples from responders (blue bars).

Given the strong association between microbiome composition and response to immunotherapy as seen previously in other cancers like melanoma (34–36) we sought to measure differences in the urinary microbial composition between pT0 Trial bladder cancer patients who showed complete response to neoadjuvant chemotherapy and those who did not. **Figure 5A** shows the microbial composition in each patient from both groups. There were no statistically significant differences in alpha diversity between responders and nonresponders (**Figure 5B**). PCoA did not reveal significant differences in the beta diversity of samples collected from responders and non-responders (**Figure 5C**). At the genus level, relative abundance of taxa showed differences between the two groups (**Figure 5D**) with *Granulicatella* (p<0.0001) and *Proteus* (p<0.001) enriched in non-responders (**Figure 5E**). LDA scores revealed abundance of *E. Faecalis* in responders (**Figure 5F**). Not enough patients received a gemcitabine-containing regimen to determine if Gammaproteobacteria presence was associated with response.

### 3.6 Effect of antibiotic treatment on urine microbiome in human samples

**Figure 6:**
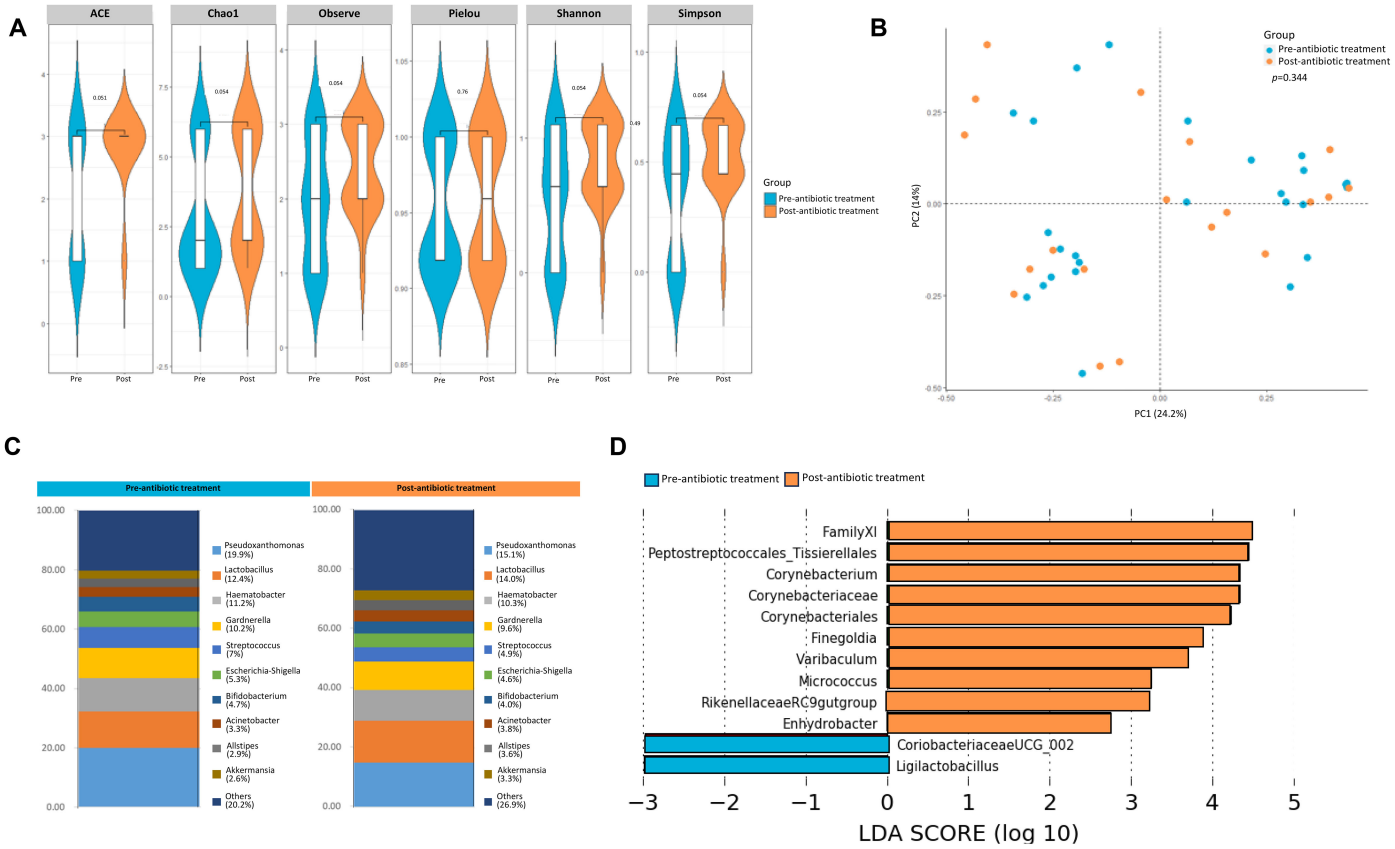
Effect of antibiotic treatment on the urine microbiome. **(A)** Genus-level alpha diversity plots of the urine microbiome from samples taken from healthy controls pre- and post antibiotic treatment with error bars representing variability in diversity scores **(B)** PCoA using weighted unifrac distances of beta diversity stratified by antibiotic treatment (ADONIS; Bray-Curtis; *p*=0.344) **(C)** Bar plot showing bacterial composition at the genus level. Relative abundance is plotted for taxa from each group. **(D)** Histogram of LDA scores for differentially over-abundant taxa in urine samples from urine samples pre-(blue bars) and post-antibiotic treatment (orange bars).

Antibiotic prophylaxis and therapy remain crucial in successfully managing various urological diseases, including infections, diagnostic procedures, surgical interventions, and transplants. Preprocedure antibiotics are required by the Department of Human Services in order for payment to occur to healthcare facilities and providers. As such, the urinary microbiome may be iatrogenically affected, but the effect of preoperative antibiotics has not been assessed. Further, conventional antibiotic therapy causes long-term alterations in the urinary microbiome, chronic recurring cystitis, and the development of multidrug resistant pathogens, often relating to prolonged episodes of infection (28). We sought to determine the effect of antibiotics on the urinary microbiome using samples collected from patients undergoing non-endourological procedures, as insertion of a foreign body into the urethra/bladder could confound the analysis. There was no statistically significant change in either alpha or beta diversity one month post antibiotic treatment (**Figures 6A, B**). Differential abundance was seen between taxa at the genus level pre- and post- antibiotic treatment (**Figure 6C**). Several alpha diversity metrics approached significant difference thresholds (5 of 6 metrics had p value of 0.051 or 0.054). This probably reflects a small sample size. LDA analysis, however, did reveal differential enrichment of taxa like *Corneybacterium*, *Finegoldia*, *Varibaculum* in samples obtained post antibiotic treatment (**Figure 6D**), which is consistent with the fastidiousness of Gram-positive organisms.

### 3.7 Similarities in microbiome between urine and tissue samples

**Figure 7:**
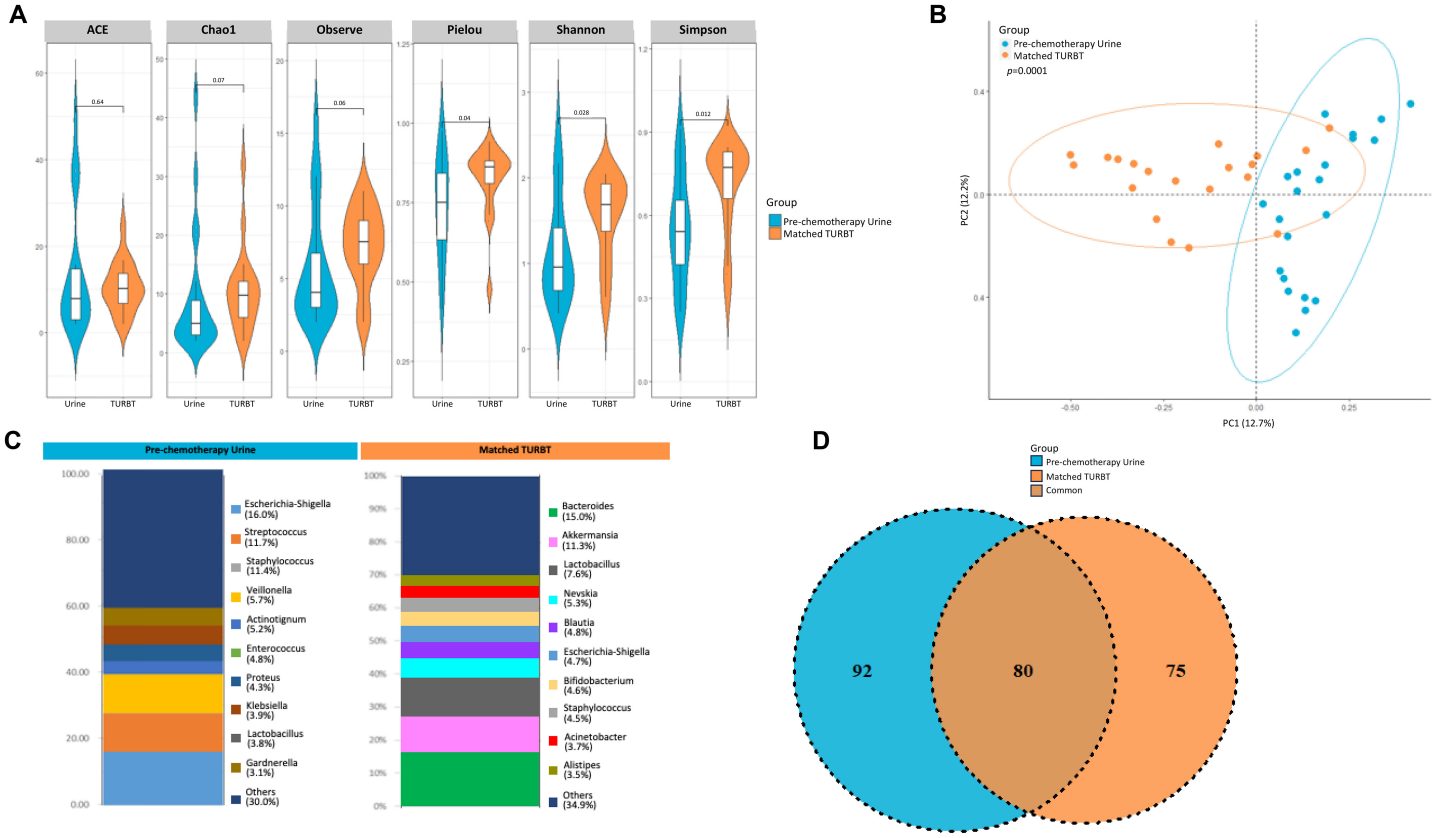
Variability in microbiome between urine and matched TURBT samples from bladder cancer patients. **(A)** Genus-level alpha diversity plots of the urine microbiome from urine samples and matched TURBT samples from bladder cancer patients with error bars representing variability in diversity scores **(B)** PCoA using weighted unifrac distances of beta diversity stratified by sample type (ADONIS; Bray-Curtis; *p*=0.0001) **(C)** Bar plot showing bacterial composition at the genus level. Relative abundance is plotted for taxa from each group. **(D)** Venn diagram showing number of shared and unique OTUs between urine and matched TURBT samples from the bladder cancer patients.

Matched diagnostic TURBT samples was also collected from the patients enrolled in the pT0 Trial, in addition to the urine samples collected pre- and post- chemotherapy. We sought to identify similarities/differences in the microbial composition between the pre-chemotherapy urine and TURBT samples from bladder cancer patients. Though the alpha diversity metrics measured by ACE, Chao1, and observed species were not significantly different between pre-chemotherapy urine and TURBT samples, we did observe a significant difference in evenness (Pielou, *p=*0.04) and diversity indices (Shannon, p=0.03; and Simpson, *p*=0.01), with the TURBT samples showing higher diversity and evenness compared to the urine samples (**Figure 7A**). PCoA analysis also revealed a clear separation between the microbial composition seen in urine and TURBT samples (*p*<0.001, **Figure 7B**). Distribution of each genus in the prechemotherapy urine and matched tissue is illustrated in **Figure 7C**, with *Escherichia* being the most predominant single genus in urine but *Bacteroides* being the most predominant genus in tissue. In comparing similarities and differences, 80 taxa were found to be common between the two groups with 92 and 75 taxa unique to the pre-chemotherapy urine samples and TURBT samples, respectively (**Figure 7D**).

### 3.8 Alteration of the tissue microbiome during murine carcinogen treatment

**Figure 8:**
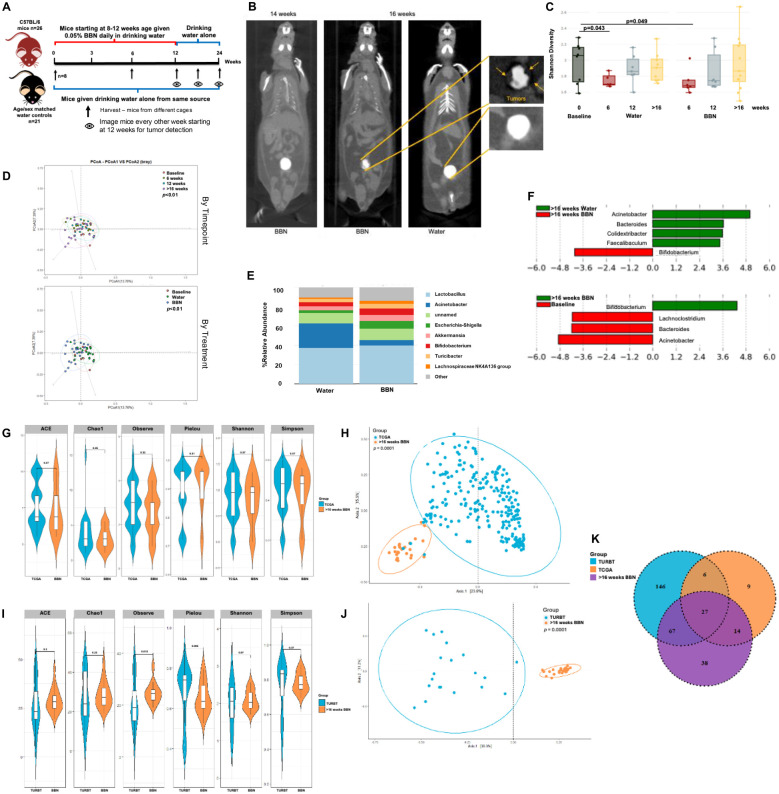
Microbiome alterations in mice over the course of tumor development: **(A)** Schematic showing treatments in the two groups of mice **(B)** µCT excretory urography images of mice demonstrating filling deficit indicated by yellow arrows in tumor-bearing mouse at week 16 **(C)** Shannon diversity plot depicting microbiome diversity at baseline, 6, 12 and post 16 weeks in both mice on water alone and with BBN treatment **(D)** PCoA using weighted unifrac distances of beta diversity stratified by timepoint (top) and by treatment (bottom) (ADONIS; Bray-Curtis; top, *p*<0.01; bottom, *p*<0.01) **(E)** Bar plot showing bacterial composition at the genus level in the 2 groups of mice **(F)** Histogram of LDA scores for differentially abundant taxa in tumor samples from water vs BBN mice at the post 16 weeks timepoint (top) and between post tumor BBN mice vs baseline (bottom). **(G)** Genus-level alpha diversity plots of TCGA samples compared to tumor samples taken at >16 weeks from mice treated with BBN with error bars representing variability in diversity scores **(H)** PCoA using weighted unifrac distances of beta diversity stratified by sample type (ADONIS; Bray-Curtis; *p*=0.0001) **(I)** Genus-level alpha diversity plots of TURBT samples compared to tumor samples taken at >16 weeks from mice treated with BBN with error bars representing variability in diversity scores **(J)** PCoA using weighted unifrac distances of beta diversity stratified by sample type (ADONIS; Bray-Curtis; p=0.0001) **(K)** Venn diagram illustrating the number of shared and unique operational taxonomic units (OTUs) between TCGA samples, TURBT samples, and tumor samples from mice treated with BBN.

We also studied bladder microbial changes in mice as they undergo tumorigenesis in response to BBN (*N*-Butyl-N-(4-hydroxybutyl)nitrosamine) exposure. BBN is a metabolite found in tobacco smoke and is the most widely used carcinogen-induced animal model of bladder cancer. This model accurately represents histological and genetic features of human bladder tumors (37, 38). Bladder tissue samples were collected from mice at 6 weeks, 12 weeks and upon tumor formation as assessed by µCT excretory urography in mice that were given 0.01% BBN in their drinking water (**Figures 8A, B**). BBN was given till week 12 after which, mice were switched to regular drinking water. Age and sex-matched mice were given drinking water control from the same water source. Shannon diversity metric showed significant differences in microbial populations between baseline and 6 weeks of BBN treatment (p<0.05). However, similarities were also observed in mice that were given drinking water alone suggestive of change in background flora during this time (**Figure 8C**). Beta diversity measured by Bray Curtis dissimilarity index demonstrated that the bladder microbial composition significantly differed by timepoint (p=0.002) and treatment (p=0.004; **Figure 8D**). At genus level, differences in bacterial composition between the 2 groups of mice could be observed (**Figure 8E**). LefSe analysis showed that increased abundance of *Bifidobacterium* (p<0.05) was associated with tumor presence. At the same time, genera *Faecalibaculum*, *Acinetobacter* and *Bacteroides* were associated with healthy bladder (**Figure 8F**). No differences were observed in alpha-diversity metrics between male and female mice within both treatment groups (data not shown). However, within the group of female mice, there was an overall trend of reduction in the diversity in the ‘BBN’ groups compared to water control while these differences do not exist in male mice (data not shown).

## 4 Discussion

Key microbes that help boost the effect of existing anti-cancer interventions have been identified (39, 40). The bladder cancer microbiome might be similarly constructed to facilitate response, especially given the high response rates to chemotherapy and local and systemic immunotherapies. After being initially thought sterile, the urine is now considered a reservoir for several commensals that may consist of varying composition based on gender, age, diet and differences in health and disease (11, 14). These microbial populations may very well contribute to the onset of bladder cancer and can be manipulated to help in the prevention, early detection and treatment of the disease. We sought to characterize the urinary microbiome of bladder cancer patients and compare any changes that may have resulted upon neoadjuvant chemotherapy.

Analyzing previously published TCGA data from the bladder cancer cohort, we were able to identify specific viral and bacterial species that were preferentially enriched in the tumors when compared to blood. These bacterial populations include *B. thetaiotamicron, A. mucinaphila* and *Bifidobacterium* spp. that are positively associated with response to anti-CTLA4 (25) and anti-PD-L1 (26) therapies in cancer patients and preclinical models. Studies have postulated that the presence of these bacteria in the gut has a distinct effect on both the innate and adaptive immune response to tumor through the increase in T helper-cell response, decreased number of regulatory T cells and increased number of antigen-presenting dendritic cells (41). Given the high response rate of checkpoint inhibitor therapy in neoadjuvant and metastatic settings for bladder cancer patients (42–45), the presence of these bacterial species in tissue itself is noteworthy. In keeping with already published data on differences in bacterial diversity and composition according to gender (12, 13), we also found that the bacterial genera *Lactobacillus* and *Prevotella* alongside *A. prevotii* and *F. magna* were enriched in female bladder cancer patients. Additionally, we found that the bacterial communities were distinctly associated with different molecular sub-types with the genera *Prevotella* being more prevalent in TCGA subtypes III and IV, basal tumors. Basal tumors are more prevalent in females, so the association with female GU commensals may only be an artifact, or it is possible that the microbiome sculpts the expression subtype. One could imagine that basal and luminal cell types in the normal bladder have different microbial burden because only luminal cells directly contact urine; tumors that descend from these cell types might be reflective of those exposures. Basal tumors have been previously characterized to be sensitive to cisplatin-based chemotherapy (46). Gene set enrichment analysis showed the association of *Mycoplasma* with the Hallmark DNA repair gene set and with the KEGG pyrimidine metabolism pathway. Gemcitabine, a pyrimidine nucleoside, is detoxified in pancreatic ductal adenocarcinoma due to the activity of the long isoform of the enzyme cytidine deaminase expressed by intratumoral *Mycoplasma hyorhinis* (47). We find that a common GU pathogen metabolizes and detoxifies gemcitabine. Given the burden of Gammaproteobacter in our 16S urine analysis of bladder cancer patients (**Figure 2E**), it is plausible that these bacteria can detoxify intravesical or systemic gemcitabine which is given as treatment. Further analysis of such therapy-modulating microbes could elucidate a new mechanism of chemoresistance in bladder cancer.

To further corroborate TCGA findings and establish the urinary microbiome associated with response to neoadjuvant chemotherapy in bladder cancer patients, we characterized bacterial profiles from urine samples from patients in a clinical trial and healthy urine donors using 16S metagenomics. The urinary microbiome profiles of healthy patients had dozens of differentially enriched OTU and different community structure. With regard to OTU that were differentially abundant based on response status, *Granulicatella* and *Proteus* were enriched in non-responders while *Enterococcus*, and *Faecalis* were enriched in responders. Responders also had an increased diversity in their urine microbiome. These associations do not prove causation but with functional studies, bacterial OTUs could then be developed as therapies or therapeutic targets in the treatment of bladder cancer to enhance therapeutic response. Additionally, Gammaproteobacteria such as *E. coli* can metabolize gemcitabine, reducing its effectiveness as a chemotherapeutic agent (17, 48). We found that incubating UPEC with gemcitabine resulted in the conversion of the latter to its inactive metabolite, dFdU. Presence of such bacteria and the resultant deactivation can potentially lead to chemotherapy resistance, making it harder to achieve successful treatment outcomes.

Pre- and post-chemotherapy urine samples from the pT0 cohort had similar microbial alpha and beta diversity, however revealing enrichment of *Streptococcaceae* in samples taken post-chemotherapy. To validate the results obtained using 16S rRNA sequencing, we compared pre-chemotherapy urine samples from the pT0 cohort to whole-genome sequencing results from a smaller cohort of samples from the RETAIN trial. Large proportion of OTUs were commonly identified by both methods, suggestive of 16S sequencing data providing a reasonable representation of the dominant bacterial taxa (**Supplementary Figure 2**). Another interesting observation was the differential make-up of microbial composition between pre-chemotherapy urine and matched TURBT samples. Based on data presented herein, the urinary microbiome shares about 32% of the microbiome with the TURBT samples. Previous two studies comparing tissue and urine microbiome in bladder cancer patients have shown conflicting results (39, 40); Pederzoli showed that the urine and tissue microbiome shared 80% of taxa in 49 subjects, but Mansour showed that there was less overlap in only 4 subjects. Methodological differences may account for some of the discrepancies.

Antibiotic use by bladder cancer patients prior to surgery may be a confounding factor due to its effect on the microbiome. Antibiotic administration prior to all surgical procedures to reduce post-operative risk of infections is mandated by Centers for Medicare/Medicaid Services to avoid financial penalties (49). Additionally, despite the administration of peri-operative antibiotics prior to sampling in TCGA, presence of diverse microbiomes were still present. Whether these represent live, culturable taxa can be debated. It is possible that the genetic material was detected from dead bacteria. However, we were able to corroborate with our study, that prophylactic use of antibiotics (single dose) did not significantly alter the urinary microbiome at the timepoints analyzed. Antibiotic treatment (e.g. 7-day treatment with renally excreted antibiotic for genitourinary infection) are more likely to have a longer lasting effect but doing that sort of study with matched samples pre/post spontaneous infection would be difficult logistically.

Little is known about microbial changes that occur during bladder carcinogenesis, so we measured changes occurring during the course of BBN carcinogenesis in mice using 16S metagenomics. Significant differences in alpha-diversity metrics in BBN mice at 6 weeks compared to control dissipated with the cessation of treatment at 12 weeks, suggesting a restoration of microbial community upon BBN removal despite the carcinogenesis cascade having started. These results provide an initial understanding of microbiome alterations in the bladder during the process of BBN-induced tumorigenesis, however further definitive studies in both mice and human are needed to confirm these findings. Comparing microbial signatures obtained in mice that had tumors, with microbes identified in the TCGA analysis and from TURBT samples, a larger overlap was seen between OTUs identified in BBN mice and the TURBT samples (**Figures 8G–K**). Beta-diversity metrics reveal largely different bacterial compositions when comparing BBN mice and the TCGA/TURBT samples. This could be due to different sampling techniques between the three sources or the inherent differences between mice and humans that must be considered when translating findings to human.

In all, we have described the bladder cancer microbiome in several key and relevant settings. This study confirms previous associations between bacteria and identifies new relationships, especially between bacteria known to impact therapeutic effectiveness of common anti-bladder cancer therapies. These findings can be the basis of additional future studies.

## Data Availability

The original contributions presented in the study are publicly available. This data can be found here: https://www.ncbi.nlm.nih.gov/bioproject/PRJNA1261646.

## Supplementary material

Supplementary Figure 1 Analysis of urine microbiome differences in bladder cancer patients before and after receiving chemotherapy. Bar plots showing bacterial composition at the genus level. Relative abundance is plotted for taxa from pre and post chemotherapy urine samples from 6 patients

Supplementary Figure 2 Comparison of results obtained via 16S of the pT0 cohort and whole-genome sequencing of urine samples from the RETAIN cohort. Venn diagram illustrating the number of shared and unique operational taxonomic units (OTUs) between urine samples taken from bladder cancer patients pre-chemotherapy from the pT0 cohort and urine samples from the RETAIN cohort.
