# Supplementary material for: Bladder cancer microbiome and its association with chemoresponse
Source: Front Oncol. 2025 Jul 21;15:1506319. doi: 10.3389/fonc.2025.1506319 (PMC12318727; doi:10.3389/fonc.2025.1506319)
Supplement: Supplementary Figure 1 — Analysis of urine microbiome differences in bladder cancer patients before and after receiving chemotherapy. Bar plots showing bacterial composition at the genus level. Relative abundance is plotted for taxa from pre and post chemotherapy urine samples from 6 patients [file DataSheet1.pdf]

SUPPLEMENTAL TABLES

Supplemental Table 1. 16S PCR primer, adaptor and index sequences.

PCR stage	Forward Primers	Sequence	Reverse Primers	Sequence
Stage 1	i16SV3Ffs0	TCGTCGGCAGCGTCAGATGTGTATAAGAGACAGCCTACGGGNGGCWGCAG	i16sV4Rfs0	GTCTCGTGGGCTCGGAGATGTGTATAAGAGACAGGACTACHVGGGTATCTAATCC
	i16SV3Ffs1	TCGTCGGCAGCGTCAGATGTGTATAAGAGACAGTCCTACGGGNGGCWGCAG	i16sV4Rfs1	GTCTCGTGGGCTCGGAGATGTGTATAAGAGACATGGACTACHVGGGTATCTAATCC
	i16SV3Ffs2	TCGTCGGCAGCGTCAGATGTGTATAAGAGACAGGTCCTACGGGNGGCWGCAG	i16sV4Rfs2	GTCTCGTGGGCTCGGAGATGTGTATAAGAGACACTGGACTACHVGGGTATCTAATCC
	i16SV3Ffs3	TCGTCGGCAGCGTCAGATGTGTATAAGAGACAGAAGCCTACGGGNGGCWGCAG	i16sV4Rfs3	GTCTCGTGGGCTCGGAGATGTGTATAAGAGACAACTGGACTACHVGGGTATCTAATCC
Stage 2	ii16SV3FA	AATGATACGGCGACCACCGAGATCTACACTAGATCGCTCGTCGGCAGCGTC	ii16SV4R1	CAAGCAGAAGACGGCATACGAGATTCGCCTTAGTCTCGTGGGCTCGG
	ii16SV3FB	AATGATACGGCGACCACCGAGATCTACACCTCTCTATTCGTCGGCAGCGTC	ii16SV4R2	CAAGCAGAAGACGGCATACGAGATCTAGTACGGTCTCGTGGGCTCGG
	ii16SV3FC	AATGATACGGCGACCACCGAGATCTACACTATCCTCTTCGTCGGCAGCGTC	ii16SV4R3	CAAGCAGAAGACGGCATACGAGATTTCTGCCTGTCTCGTGGGCTCGG
	ii16SV3FD	AATGATACGGCGACCACCGAGATCTACACAGAGTAGATCGTCGGCAGCGTC	ii16SV4R4	CAAGCAGAAGACGGCATACGAGATGCTCAGGAGTCTCGTGGGCTCGG
	ii16SV3FE	AATGATACGGCGACCACCGAGATCTACACGTAAGGAGTCGTCGGCAGCGTC	ii16SV4R5	CAAGCAGAAGACGGCATACGAGATAGGAGTCCGTCTCGTGGGCTCGG
	ii16SV3FF	AATGATACGGCGACCACCGAGATCTACACACTGCATATCGTCGGCAGCGTC	ii16SV4R6	CAAGCAGAAGACGGCATACGAGATCATGCCTAGTCTCGTGGGCTCGG
	ii16SV3FG	AATGATACGGCGACCACCGAGATCTACACAAGGAGTATCGTCGGCAGCGTC		
	ii16SV3FH	AATGATACGGCGACCACCGAGATCTACACCTAAGCCTTCGTCGGCAGCGTC		

SUPPLEMENTAL FIGURES

Supplementary Figure 1: Analysis of urine microbiome differences in bladder cancer patients before and after receiving chemotherapy.

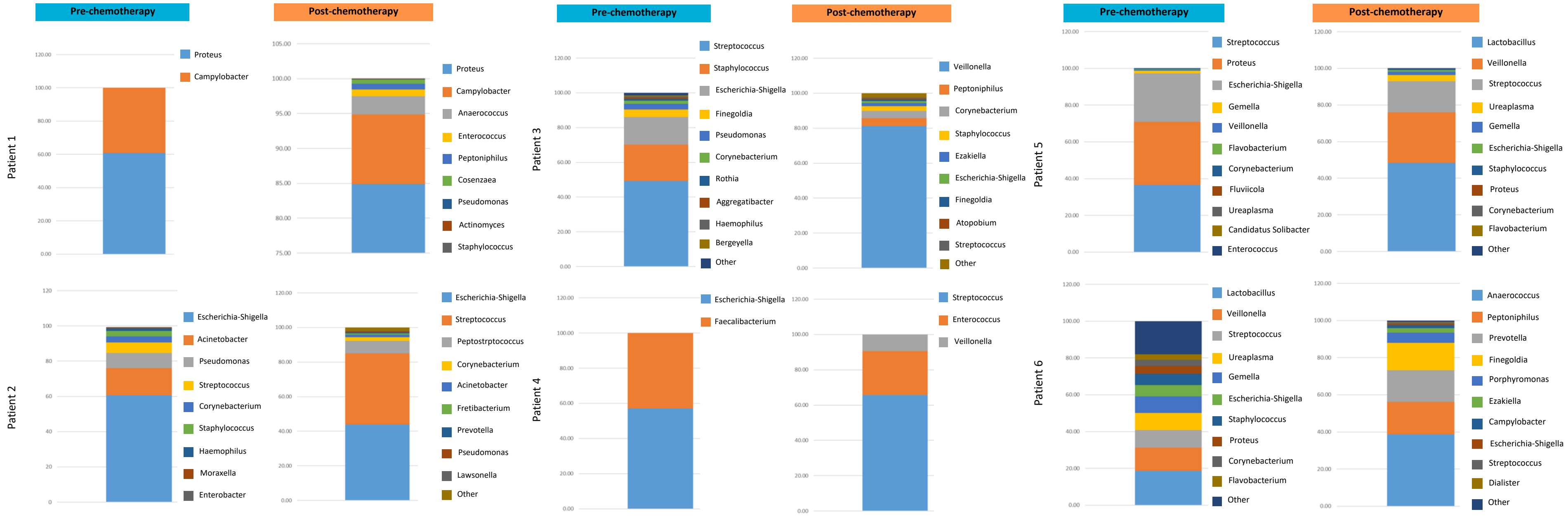

Bar plots showing bacterial composition at the genus level. Relative abundance is plotted for taxa from pre and post chemotherapy urine samples from 6 patients

Supplementary Figure 2: Comparison of results obtained via 16S of the pT0 cohort and whole-genome sequencing of urine samples from the RETAIN cohort.

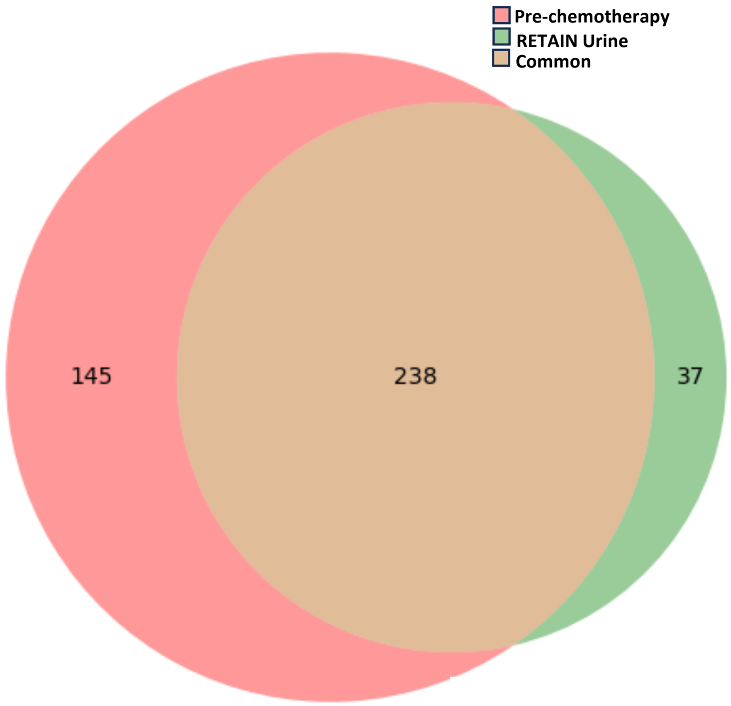

Venn diagram illustrating the number of shared and unique operational taxonomic units (OTUs) between urine samples taken from bladder cancer patients pre-chemotherapy from the pT0 cohort and urine samples from the RETAIN cohort.
